# Microbubble-Mediated Ultrasound Outweighs Low-Intensity Pulsed Ultrasound on Osteogenesis and Neovascularization in a Rabbit Model of Steroid-Associated Osteonecrosis

**DOI:** 10.1155/2018/4606791

**Published:** 2018-09-12

**Authors:** Dan-Feng Xu, Guo-Xin Qu, Shi-Gui Yan, Xun-Zi Cai

**Affiliations:** ^1^Department of Orthopaedic Surgery, Second Affiliated Hospital, School of Medicine, Zhejiang University, Jie-fang Road 88, Hangzhou, 310009, China; ^2^Department of Orthopaedic Surgery, Shaoxing Central Hospital, Hua-yu Road 1, Keqiao, Shaoxing, 312030, China

## Abstract

Microbubbles magnify the acoustic pressure of low-intensity pulsed ultrasound (LIPUS) and may enhance its bioeffect for diagnostic and therapeutic purposes. This study compared the effect of this novel microbubble-mediated ultrasound (MUS) with that of the traditional LIPUS on osteogenesis and neovascularization in a rabbit model of steroid-associated osteonecrosis. We hypothesized that MUS might outweigh LIPUS on promoting osteogenesis and neovascularization in steroid-associated osteonecrosis. The bilateral femoral head necrosis was induced by lipopolysaccharide and methylprednisolone in the rabbits. The indices of bone mineral density (BMD), trabecular number, maximal loading strength, and mineral apposition rate were analyzed, demonstrating that the animal model of steroid-associated osteonecrosis was successfully established. Both the MUS group (G_M_) and the LIPUS group (G_L_) were insonated 20 min daily for six weeks. G_M_ received an extra intracapsular injection of microbubbles before insonation every other day. Fluorescence bone labeling, Micro-CT Analysis, biomechanical test, quantitative real-time PCR, Western blot analysis, and histological evaluation were performed for comparing G_M_ with G_L_. The results demonstrated a 39% higher mineral apposition rate in G_M_ compared with G_L_. The BMD and the maximal loading strength of femoral head of G_M_ increased by 4.3% and 27.8% compared to those of G_L_, respectively. The mRNA and protein expression of BMP-2 and VEGF were also significantly higher in G_M_. The number of blood vessels of G_M_ was 65% greater than that of G_L_. MUS is more potent than LIPUS in enhancing osteogenesis, neovascularization, and biomechanical strength of femoral head in the animal model of steroid-associated osteonecrosis. Without increasing the intensity of insonation or the risk of tissue damage, MUS is better for inhibiting the process of steroid-associated osteonecrosis.

## 1. Introduction

Steroid usage is the most common nontraumatic cause of osteonecrosis [1]. Up to 40% of steroid users will develop some degree of osteonecrosis, known as the steroid-associated osteonecrosis [2, 3]. The precise mechanism in which corticosteroids induce osteonecrosis is currently unclear. Hypotheses include fat cell hypertrophy, fat embolization, intravascular coagulation, and osteocyte apoptosis [4–6]. To our knowledge there is no established preventative method for steroid-associated osteonecrosis.

Low intensity pulsed ultrasound (LIPUS) is understood as a noninvasive, feasible, and economic modality for the delayed union and the nonunion of bone fractures. It is approved by the Food and Drug Administration in 1994. Since then, the research exploring the prospects of its clinical application has not stopped. The therapeutic application of LIPUS is worldwide nowadays. Recently, our team for the first time reported the effect of LIPUS on steroid-associated osteonecrosis in a rabbit model of steroid-associated osteonecrosis [7]. The authors revealed that LIPUS could promote the bone repair and the vascularization* in vivo *[7, 8]. It provides us a new insight to preventing the progression of steroid-associated osteonecrosis.

Microbubbles have been increasingly used to improve the therapeutic effect of LIPUS [9]. Microbubbles, with a gas-filled structure, are stabilized by a protein, lipid, or polymer shell. Microbubbles are initially developed as contrast agents for ultrasound imaging [10, 11]. They have the ability to produce mechanical actuations and shockwaves when they interact with the focal zone of LIPUS. More and more therapeutic applications of microbubbles are emerging at present [9, 12–14]. This novel ultrasound, known as microbubble-mediated ultrasound (MUS), has the ability to produce greater microstream and microjet than LIPUS [15, 16]. To our knowledge, whether MUS has more effect on the repair progress in steroid-associated osteonecrosis is unknown.

We hypothesized that MUS might outweigh the repair process of femoral head in steroid-associated osteonecrosis more efficiently than LIPUS. This study aimed to explore the enhancive effect of MUS on bone microstructure, mechanical strength, and vascularization compared with LIPUS

## 2. Methods and Materials

### 2.1. Experimental Design

**Figure 1:**
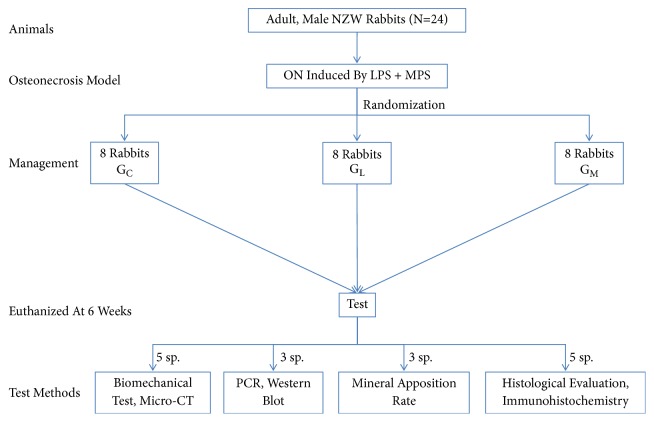
The flowchart of the study design (reproduced from Zhu et al. (2015), under the Creative Commons Attribution License/public domain). NZW = New Zealand White rabbits; ON = osteonecrosis; LPS = Lipopolysaccharide; MPS = methylprednisolone; G_C_ = the control group; GL = the LIPUS group; GM = the MUS group; sp. = specimen.

The experimental protocol was approved by the Animal Care and Use Committee of Zhejiang University. Twenty-four adult male New Zealand rabbits (mean weight: 2.69 kg, range 2.45 to 3.00 kg) were used. The rabbit model of steroid-associated osteonecrosis was prepared using a protocol similar to the previous studies [17, 18]. To begin with, all the rabbits received intravenous injections of 10 *μ*g/kg body weight of lipopolysaccharide (Escherichia coli 0111:B4; Sigma, St Louis, MO, USA). After 24 hours three injections of methylprednisolone (Pharmacia and Upjohn, Puurs, Belgium) (20 mg/kg body weight) were given intramuscularly every 24 hours. It has been reported that the osteonecrosis lesions began after two weeks and completed after six weeks [17]. The rabbits were randomly divided into three groups: the control group (G_C_), the LIPUS group (G_L_), and the MUS group (G_M_)(n = 8). Both G_L_ and G_M_ received LIPUS. Additionally G_M_ received a 0.1 ml intracapsular injection (guided by B-ultrasound) of sulfur hexafluoride-filled microbubble solution (volume concentration: 7-8 *μ*l/ ml, mean diameter: 2.5 mm, 90%, 6.0 mm, 99%, and 11.0 mm; Sonovue, Bracco, Geneva, Switzerland) before insonation [9] (Figure 1). Three rabbits were randomly selected for MRI (T1-weighted image, TR 400 ms, TE 20 ms; T2-weighted image, TR 2000 ms, and TE 106 ms) two weeks after methylprednisolone injection to test whether osteonecrosis was induced.

### 2.2. Insonation

LIPUS (US 13; Cosmogamma Co, Bologna, Italy) was used at a pulse frequency of 1.0 MHz, an output intensity of 200 mW/cm^2^, a 20% duty cycle, and a 100-Hz repetition rate [7]. Microbubble solution was prepared according to the manufacturer's instructions. The skin around the hip was cleaned and ultrasound gel (KL-250; Keppler Co, Hangzhou, China) was used to couple the LIPUS transmission. The rabbits were anesthetized with 3% pentobarbital sodium before insonation. LIPUS therapy was administered to the bilateral femoral head 24 hours after the lipopolysaccharide injection and before the first methylprednisolone injection. The sham insonation was administered to the rabbits of G_C_ (the device was turned off). In this study, rabbits were insonated with 20 minutes per day for six weeks. Then all the rabbits were euthanized with an overdose intravenous injection of pentobarbital sodium. Bilateral femurs of the hind leg were collected for analyses.

### 2.3. Fluorescence Bone Labeling

All the rabbits received intramuscular injections of a fluorescence tracer, alizarin complexone (30 mg/kg, Merck, Darmstadt, Germany), and calcein (10 mg/kg, Sigma, St. Louis, MO, USA) at 14 and 4 days, respectively, before the euthanasia to label newly mineralized bone. The mineral apposition rate (*μ*m/day) (MAR = mean vertical distance between two fluorescence bands/injection interval), which generally indicated the mineralization rate of the newly formed bone, was analyzed [19].

### 2.4. Micro-CT Analysis

The proximal parts of the bilateral femur were prepared for micro-CT (eXplore Locus SP; GE Co, Fairfield, CT, USA) scanning, with a spatial resolution of 45 *μ*m according to the protocol for animal studies. To evaluate the degree of plate-like and rod-like trabecular bone in the femoral head, a predetermined bone cylinder was used as the region of interest (3.0 mm diameter and 1.5 mm height) (Figure 4(d)) to quantify the mean volumetric bone mineral density (BMD) (mg/cc) and the trabecular architectural parameters, including the bone tissue volume density (bone volume/total volume, %), the trabecular number (/mm), the trabecular thickness (*μ*m), the trabecular spacing (*μ*m), and the connectivity density (/mm^3^) [20].

### 2.5. Biomechanical Evaluation

The loading strength of the femoral head was measured using an indentation test with a 2.8-mm-diameter indenter. The proximal femur was fixed and placed on a metal platform of a static materials tester (Z2.5; Zwick / Roell, Ulm, Germany). Alignment of the specimen was adjusted in accordance with the stainless steel rod in the upper holding device. The indenter was displacement-controlled at a constant rate of 5 mm/min and was stopped when the load markedly decreased. The maximum loading strength (N)was automatically record (TestXpert II; Zwick / Roell) [7] (Figure 5).

### 2.6. RNA Extraction and Quantitative Real-Time

mRNA levels of samples were determined by quantitative real-time PCR. In brief, the RNA of the samples were extracted with Trizol reagents (Invitrogen, USA). The primers (Table 1) were designed by Oligo 6.0 primer design software (Molecular Biology Insights, Colorado Springs, CO, USA) and synthesized by KeDuo (Hangzhou, China). Quantitative real-time PCR reactions were carried out by using a SYBR^®^ Premix Ex Taq™ Kit (TaKaRa Biotech Co, Shiga, Japan). The cycle threshold (Ct) was measured for each mRNA in each sample. The relative quantifications of the gene expressions (BMP-2 and VEGF) were calculated by the formula 2^ΔΔCT^*∗*10^5^. ΔΔCt = ΔCt_1_-ΔCt_2_.ΔCt_1_ is the value of the housekeeping gene 18S and ΔCt_2_ indicates the Ct value of the target gene [21, 22].

### 2.7. Western Blot Analysis

Protein was isolated with radioimmunoprecipitation assay buffer lysis buffer (Sangon Biotech Co, Ltd). Sodium dodecyl sulfatepolyacrylamide gel (Keduo, Hangzhou, China) was formulated and loaded and electrophoresis occurred prior to transferring to a membrane (Sangon Biotech Co, Ltd), which was closed with skimmed milk powder. The samples were reacted with the primary *β*-actin antibody, secondary antibody (VEGF and BMP-2, Goat anti-rabbit, Cruz Biotech, Inc, Santa Cruz, CA, USA), and chemiluminescence was performed. The images were analyzed with Bandscan 5.0 (Glyko Inc, Novato, CA, USA) to calculate the intensity of the bands.

### 2.8. Histologic Evaluation and Immunohistochemistry

Specimens for histological evaluation were fixed in a 4% paraformaldehyde solution. After decalcification in 10% EDTA, the samples were embedded in paraffin. Five-micron sections were cut and stained with hematoxylin and eosin for evaluation of necrosis and calculation of fat cell size. The *α*-smooth muscle actin staining was used to assess the number and the mean diameter of blood vessels [23].

The slices were examined using a light microscope (Olympus BX51, Tokyo, Japan). Five fields in the subchondral area of the femoral head on each section were chosen [7]. The first field was located at the approximate center of the femoral head and the other four were located on both sides of the first field. The following parameters were assessed: the average diameter of the fat cells [7, 17] and the number and the diameter of blood vessels. Different fields per slice were converted to digital pictures and quantitative analysis was performed using Image-Pro Plus 6.0 (Media Cybernetics Inc, Rockville, MD, USA).

### 2.9. Statistical Analysis

The data were expressed as the mean ± standard deviation. SPSS 20.0 software (SPSS, Chicago, IL, USA) was used to analyze the data by one-way analysis of variance with Scheffe's multiple comparison method. p < 0.05 was considered statistically significant.

## 3. Results

### 3.1. MRI Two Weeks after Methylprednisolone Injection

**Figure 2:**
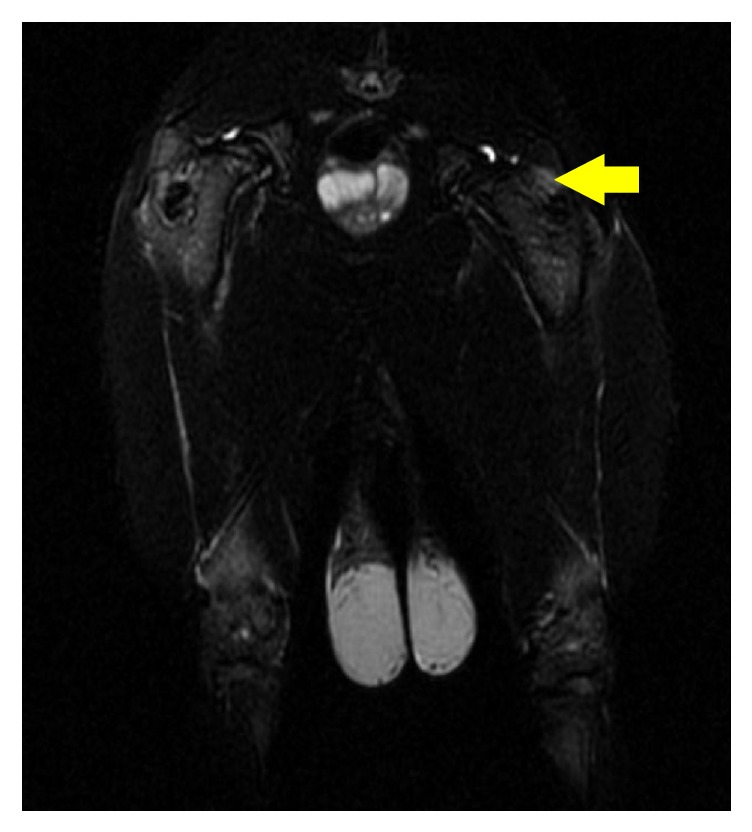
The edema sign (the yellow arrow) was observed in the proximal femur.

The image showed the appearance of the mild edema in the proximal femur (Figure 2).

### 3.2. Mineral Apposition Rate Evaluation

**Figure 3:**
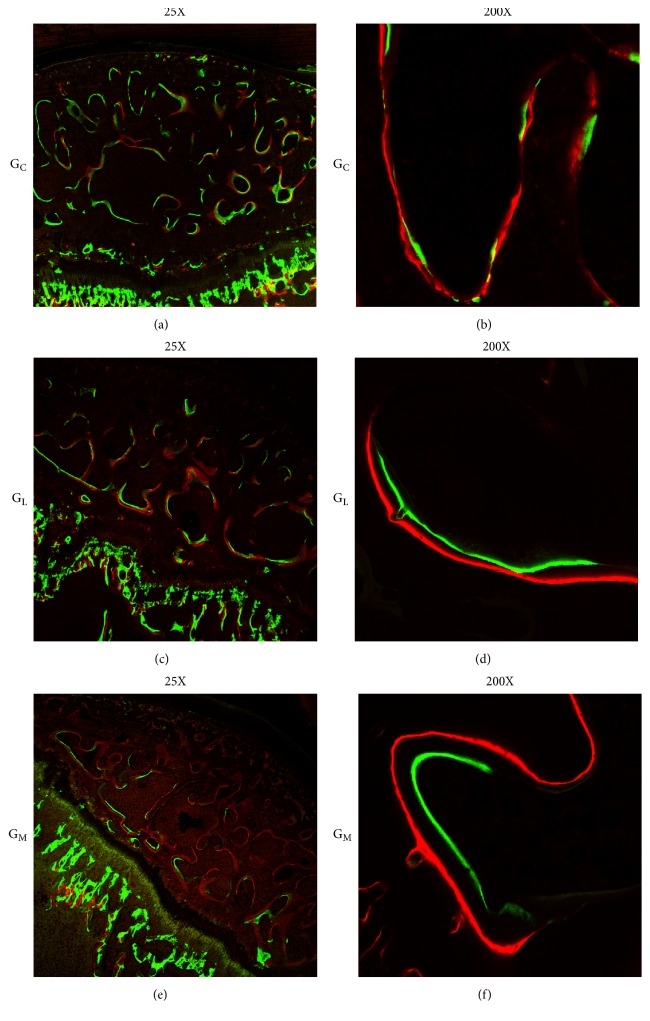
The bone of femoral head was labeled during bone regeneration and remodeling by calcein (green) and alizarin complexone (red). Representative labeling images from each group. The capacity of bone regeneration was the strongest in G_M_ (f).

The mineral apposition rate of the femoral head was detected by the distance between the parallel fluorescent bands. The two fluorescent labels (green bands, calcein; red bands, alizarin complexone) were clear (Figure 3). According to the statistical analysis, there was a significant difference in the mineral apposition rate between G_M_ (2.660 ± 0.2390 *μ*m/d) and G_L_ (1.913 ± 0.1457 *μ*m/d) (p=0.008; Table 2).

### 3.3. Osteogenesis in Femoral Head

**Figure 4:**
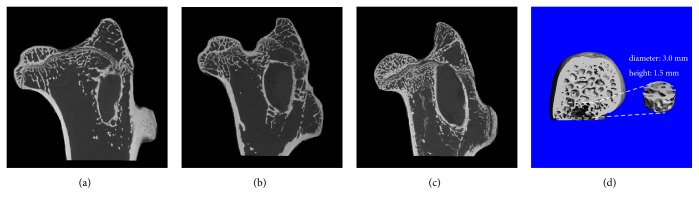
Representative micro-CT images from each group. The trabecular bone In G_M_ was thicker and denser than that in G_L_ and that in G_C_. (d) Micro-CT-based trabecular architecture and sketches of the region of interest.

Micro-CT images indicated that the trabecular bone was more compact, thicker, and denser in the subchondral bone of G_M_ (Figure 4) compared with G_L_ and G_C_. The BMD of G_M_ (932.6 ± 6.861 mg/cm^2^) was significantly higher than that of G_L_ (894.5 ± 12.84 mg/cm^2^) (p < 0.001; Table 2). The trabecular number of G_M_ (2.352 ± 0.06980 1/mm) was significantly higher than that of G_L_ (2.213 ± 0.07245 1/mm) (p = 0.039; Table 2).

### 3.4. Mechanical Strength of Femoral Head

**Figure 5:**
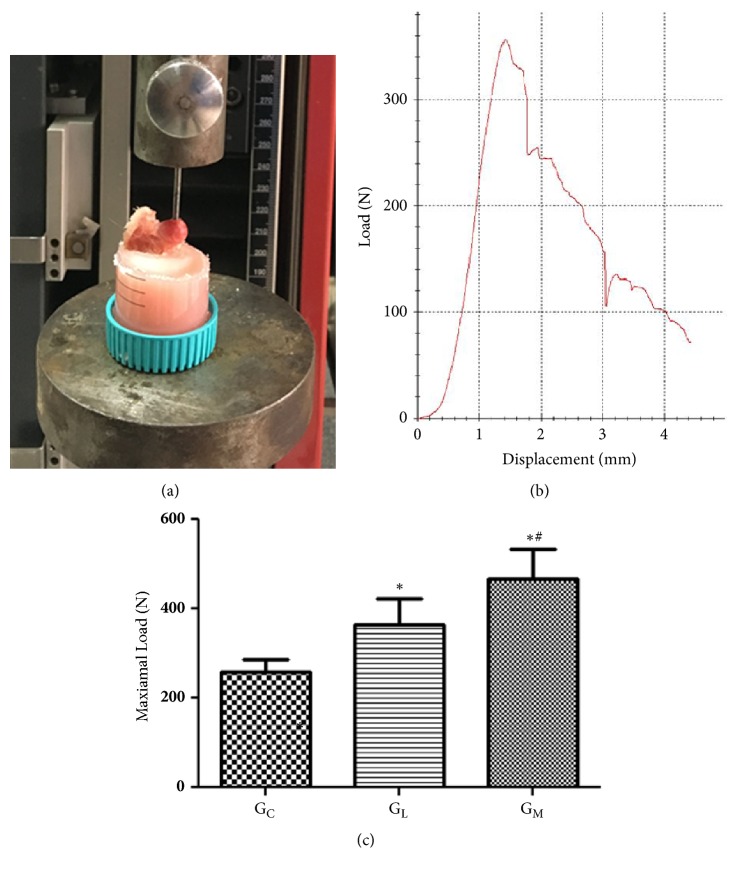
(a) Illustrating how the specimen was fixed in dental cement and the force was parallel to the long axis of the proximal femur. (b) Representative load-deformation curve obtained by the biomechanical test. (c) Statistical analyses of the biomechanical test data. *∗*p<0.05 vs. the blank group; #p<0.05 vs. G_L_.

The maximal loading strength of femoral head of G_M_ (467.2 ± 65.96 N) was significantly greater than that of G_L_ (365.6 ± 57.40 N, p < 0.001) (Figure 5).

### 3.5. BMP-2 and VEGF Expression

**Figure 6:**
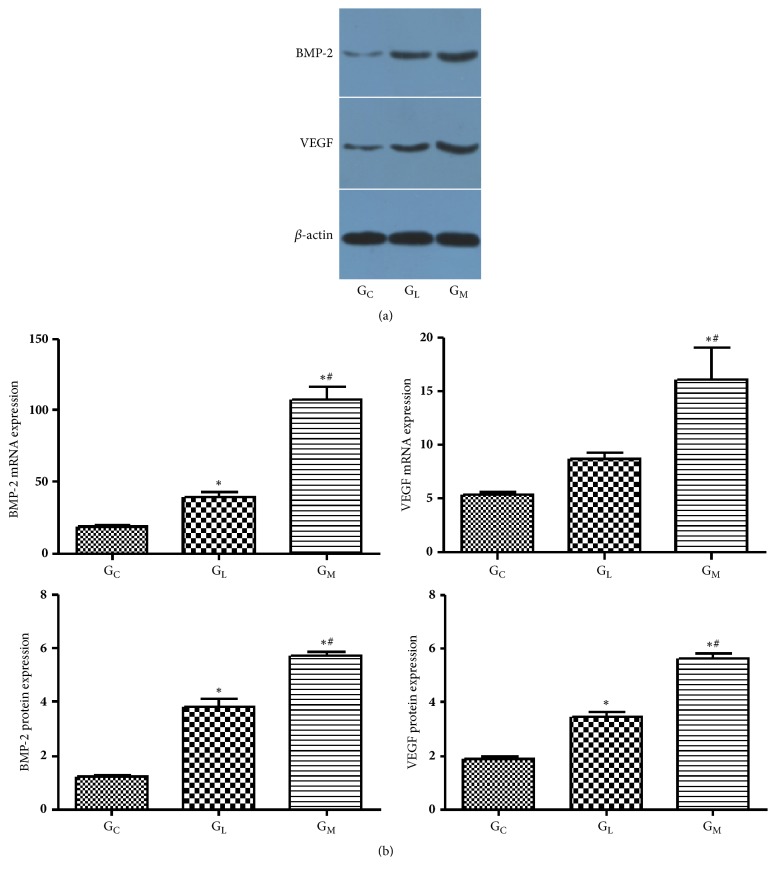
(a) These band scans showed the Western blot analysis of BMP-2 and VEGF protein expression in G_C_, G_L_ and G_M_. (b) Statistical analyses of the mRNA expressions of BMP-2 and VEGF, protein expression of BMP-2, and VEGF data. *∗*p < 0.05 vs. G_C_; #p < 0.05 vs. G_L_.

The mRNA expression of BMP-2, the protein expression of BMP-2, the VEGF mRNA expression, and the VEGF protein expression of G_M_ were significantly higher than those of G_L_ (107.7 ± 8.527 vs 39.55 ± 3.141, p < 0.001; 5.753 ± 0.1206 vs 3.860 ± 0.2706, p < 0.001; 16.09 ± 2.911 vs 8.733 ± 0.5577, p=0.006; 5.640 ± 0.2095 vs 3.467 ± 0.1656, p < 0.001; respectively) (Figure 6).

### 3.6. Histomorphometric Evaluation

**Figure 7:**
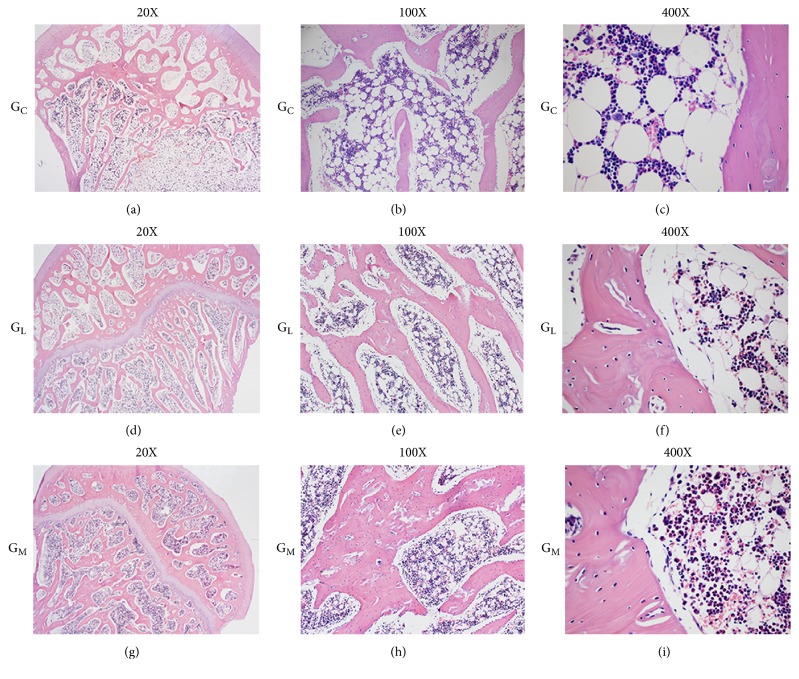
(a, b) Enlarged and massive fat cells and sparser trabecular bone were shown in G_C_. (c) The high-powered field from G_C_ showed the empty lacunae. There were smaller fat cells, thicker trabecular bone, less bone and marrow necrosis, and less empty osteocyte lacunae in G_M_ (g, h, and i) compared with that in G_C_ (a, b, and c) and that in G_L_ (d, e, and f).

The limbs of G_C_ were observed to have sparse trabeculae (Figure 7(a)) with massive empty lacunae, few osteoblasts, large marrow fat cells, and a disordered architecture of marrow tissue (Figure 7(c)). In contrast, no such changes were observed in G_M_ (Figures 7(g) and 7(i)). The average fat cell diameter of G_M_ (28.3 ± 8.4 *μ*m) was lower than that in G_L_ (34.9 ± 7.5 *μ*m) (p = 0.075, Table 2).

**Figure 8:**
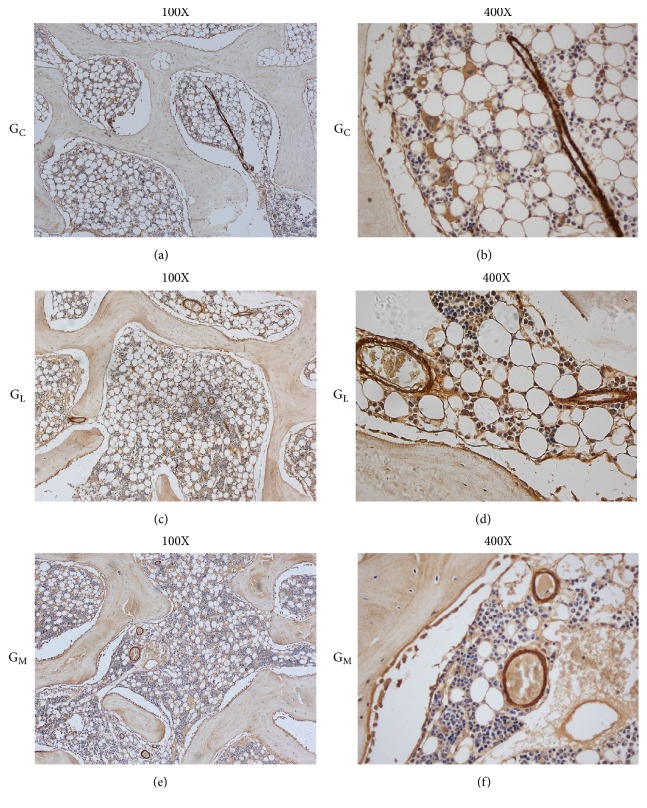
Examples of a-SMA staining from (a, b) G_C_ showed that the vessels became flattened, which were compressed by the enlarged fat cells. (e, f) The vessels in G_M_ seemed normal and more small blood vessels were observed compared with those in G_L_ (c, d).

The *α*-smooth muscle actin staining (Figures 8(a) and 8(b)) showed that the blood vessels in G_C_ were compressed by enlarged and massive fat cells. The shape of blood vessels seemed normal in G_M_. More small blood vessels were observed in G_M_ compared with G_L_ and G_C_ (Figure 8(e)). The number of vessels in G_M_ (13.7 ± 4.3 /mm^2^) was significantly higher than that in G_L_ (4.3 ± 2.2 /mm^2^, p = 0.001; Table 2). The minimal diameter of blood vessels was not statistically different (p = 0.205) between G_M_ (28.6 ± 4.6 *μ*m) and G_L_ (23.9 ± 4.2 *μ*m; Table 2).

## 4. Discussion

To our knowledge, this is the first study to investigate the bioaccoustic effect of MUS in a rabbit model of steroid-associated osteonecrosis. Our data support the hypothesis that MUS strengthens the repair process of femoral head more efficiently than LIPUS in steroid-associated osteonecrosis. The results provide radiological, biomechanical, molecular biological, and histological evidence to support our speculation that the microbubble use is a promising approach for improving the biological capabilities of LIPUS in bone repair [7].

We found that microbubble enhanced the bioaccoustic effect of LIPUS on bone repair. Our study showed that the loading strength and BMD of the proximal femoral in G_M_ was statistically higher than that in G_L_. This meant that MUS increased the biomechanical strength. Later, histopathology pictures also showed the smaller fat cells, the less bone and marrow necrosis, and the less empty osteocyte lacunae in the G_M_ compared with G_L_. We failed to find the difference of the loading strength and BMD between G_L_ and G_C_. This could be explained by the insonation duration of six weeks, which was a half of the insonation duration in our prior study [7].

The bioeffect of LIPUS, i.e., cavitation, are based on its mechanism of sonoporation [24]. Cavitation-related tissue effects arise from a complex array of interactions between microbubbles and tissue, which include the concomitant process of mechanical effects resulting from microstreaming and microbubble oscillation, growth, and collapse behaviors in the field. Sonoporation may directly increase the protein synthesis, the collagen synthesis, the membrane permeability, the integrin expression, and the cytosolic Ca^2+^ concentration [25]. Previous studies have found that the sonoporation could enhance the osteogenic differentiation and accelerated the osteoblast activity and the bone formation [26]. Our qPCR and Western blot results showed that BMP-2 expression was substantially higher in G_M_. It has been established that BMP-2 plays a central role in initiating the bone formation, stimulating the osteogenic differentiation, and increasing the osteoblast activity. The result of the mineral apposition also showed the dramatical new bone formation at the proximal of femoral head in G_M_. The data suggested that microbubbles amplified the osteogenetic effect of LIPUS.

Sufficient angiogenesis is a fundamental prerequisite of bone repair. The present study indicated that MUS had great potential in stimulating angiogenesis. Apart from the microbubble-enhanced cavitation, this bioaccoustic effect may account for the promoted production of angiogenic growth factors by MUS. Yoshida et al. [27] found the ultrasonic destruction of microbubbles delivered to the ischemic limbs recruited inflammatory cells producing VEGF. In the present study, we found that the angiogenesis was more vigorous in G_M_ compared with G_L_. Near-normal blood vessels were observed in G_M_. The minimal diameter of the blood vessels was also higher in G_M_ than that in G_L_, which could better lead to an increase in blood perfusion.

Microbubbles form endogenously in tissues with the interaction between acoustic pulses and cavitation nuclei [28]. Microbubbles are produced in limited quantities endogenously in tissue. This indicated a need for more ultrasonic energy. However the excessive energy would increase the energy absorption by tissues, resulting in a damage to these tissues. Previous literatures indicated that the cavitation could be amplified by the external microbubbles [14]. In our model, we injected 0.1 ml of microbubble solution intracapsular before insonation and exposed the proximal leg to 200 mW/cm^2^ LIPUS for 20 min per day. Depending on this acoustic pressure, microbubbles presented a stable cavitation, where microbubbles oscillated over many cycles without collapsing. The stable cavitation stimulated by microbubbles was probably responsible for the increased efficiency of LIPUS.

Limitations definitely remained. First, the duration of MUS was set at six weeks. Further studies are required to reveal the saturation point, that is, the length of time after which the bioeffect of MUS is no longer significant. Second, the osteonecrosis lesion hardly led to joint collapse in rabbits. This could be attributed to the dissimilarities in weight bearing between quadrupedal animals and bipedal humans, particularly at the hip [17, 29]. Third, the cellular and the molecular mechanisms of osteogenesis and neovascularization by MUS need further investigation.

## 5. Conclusions

The present study indicated that MUS outweighed LIPUS on promoting the osteogenesis and the neovascularization in a steroid-associated osteonecrosis model. Without increasing the intensity of insonation and the risk of tissue damage, MUS strengthened the femoral head which might prevent its further collapse. MUS holds more promise in inhibiting the pathological process of steroid-associated osteonecrosis than LIPUS.

## Figures and Tables

**Table 1:** Real-time PCR primers and conditions (reproduced from *Zhu et al.* (2015), under the Creative Commons Attribution License/public domain).

Gene	GeneBank accession	Primer sequences (5'to 3')	Size (base pairs)	Annealing (°C)
Rabbit BMP-2	NM 001082650	GGGGTGGAACGACTGGATTGTGTCTGCACGATGGCATGGTTAGT	117	64
Rabbit VEGF	AY196796	GGGGGCTGCTGCAATGATGAAAGCTGGCCCTGGTGAGGTTTGAT	97	65
Rabbit 18S	EU236696	GACGGACCAGAGCGAAAGCCGCCAGTCGGCATCGTTTATG	119	64

GeneBank available at http://www.ncbi.nlm.nih.gov/genbank.

**Table 2:** Micro-CT, biomechanical and histomorphometric evaluations, and gene expression in the three groups.

	G_C_	G_L_	G_M_
BMD (mg/cm^2^)	883.4 ± 10.28	894.5 ± 12.84	932.6 ± 6.861*∗*#
BV/TV (%)	40.30 ± 3.480	41.37 ± 4.612	41.95 ± 7.869
Tb.N (1/mm)	2.079 ±0.08272	2.213 ± 0.07245*∗*	2.352 ± 0.06980*∗*#
Tb.Th (mm)	0.1705 ± 0.01974	0.1843 ± 0.02414	0.2064 ± 0.01200*∗*
Tb.Sp (mm)	0.2672 ± 0.02893	0.2613 ± 0.02759	0.2241 ± 0.009524*∗*
Conn.D (1/ mm^3^)	10.79 ± 1.634	11.29 ± 1.053	14.39 ± 2.148*∗*#

Values are mean ± SD.

*∗*p < 0.05 vs. G_C_.

#p < 0.05 vs. G_L_.

BV/TV: bone volume fraction; Tb.N: trabecular number; Tb.Th: trabecular thickness; Tb.Sp: trabecular separation; Conn.D: connective density.

## Data Availability

The data used to support the findings of this study are included within the article.
